# c-Fos expression in the paternal mouse brain induced by communicative interaction with maternal mates

**DOI:** 10.1186/s13041-014-0066-x

**Published:** 2014-09-11

**Authors:** Jing Zhong, Mingkun Liang, Shirin Akther, Chiharu Higashida, Takahiro Tsuji, Haruhiro Higashida

**Affiliations:** Kanazawa University Center for Child Mental Development, Kanazawa, 920–8640, Japan; Department of Basic Research on Social Recognition, Research Center for Child Mental Development, Kanazawa University, Kanazawa, 920-8640 Japan

**Keywords:** Parental behavior, Retrieval, c-Fos, Mate-dependent paternal behavior, Medial preoptic area, Mice

## Abstract

**Background:**

Appropriate parental care by fathers greatly facilitates health in human family life. Much less is known from animal studies regarding the factors and neural circuitry that affect paternal behavior compared with those affecting maternal behavior. We recently reported that ICR mouse sires displayed maternal-like retrieval behavior when they were separated from pups and caged with their mates (co-housing) because the sires receive communicative interactions via ultrasonic and pheromone signals from the dams. We investigated the brain structures involved in regulating this activity by quantifying c-Fos-immunoreactive cells as neuronal activation markers in the neural pathway of male parental behavior.

**Results:**

c-Fos expression in the medial preoptic area (mPOA) was significantly higher in sires that exhibited retrieval behavior (retrievers) than those with no such behavior (non-retrievers). Identical increased expression was found in the mPOA region in the retrievers stimulated by ultrasonic vocalizations or pheromones from their mates. Such increases in expression were not observed in the ventral tegmental area (VTA), nucleus accumbens (NAcc) or ventral palladium (VP). On the following day that we identified the families of the retrievers or non-retrievers, c-Fos expression in neuronal subsets in the mPOA, VTA, NAcc and VP was much higher in the retriever sires when they isolated together with their mates in new cages. This difference was not observed in the singly isolated retriever sires in new cages. The non-retriever sires did not display expression changes in the four brain regions that were assessed.

**Conclusion:**

The mPOA neurons appeared to be activated by direct communicative interactions with mate dams, including ultrasonic vocalizations and pheromones. The mPOA-VTA-NAcc-VP neural circuit appears to be involved in paternal retrieval behavior.

## Background

Parental care is critically important for the survival and proper development of young mammals [1]. Numerous studies on the neural circuitry of female parental behavior have shown that medial preoptic area (mPOA) is important for the control of maternal behavior in rodents [2–10]. Far fewer reports investigating paternal behavior have revealed that the mPOA is responsible for paternal behavior [11–17]. Several investigations have explored the roles of the ventral tegmental area (VTA), the nucleus accumbens (NAcc) and the ventral palladium (VP) in the regulation of parental behavior [8,10,11,16,18,19]. Briefly, mPOA neurons activate VTA dopaminergic neurons, which lead to activation of the D1 receptors in NAcc. When NAcc GABAergic neurons are activated, they relieve the tonic inhibition of VP neurons. Disinhibiting VP neurons may actuate the motor activities of maternal parental behavior [2,18].

We developed a co-housing paradigm to induce paternal behavior [20–22]. When mouse sires of the ICR strain are continuously housed with their pair mates and pups for three to five days after parturition, the sires exhibit features of normal maternal care (crouching, licking and pup retrieval). When separated from their pups for 10 min and co-housed with their mates or kept in a vacated family cage during the separation, the sires exhibited retrieval behavior when reunited with their pups [20]. The sires housed alone in a new cage during the separation period for 3–30 min did not exhibit retrieval behavior upon reunion with their pups. The acquisition of retrieval behavior by the co-housed male parents required at least 5–10 min of separation, during which it is hypothesized that communicative interaction occurs between the couple [20].

We revealed that male parental care behavior was triggered by olfactory and specialized, 38-kHz ultrasonic auditory signals (USV, ultrasonic vocalization) from the dam during the interactive co-housing period [20]. The neural circuit responsible for this particular paternal behavior in the ICR strain is less clear. We recently demonstrated that scopolamine, a muscarinic receptor antagonist and muscarinic receptor-coupled KCNQ/Kv7 potassium channel opener, inhibits paternal behavior [21]. We demonstrated that paternal behaviors, including pup retrieval, grooming, crouching or huddling, were improved in CD38 knockout sires treated with oxytocin and/or with local re-expression of CD38 in the NAcc compared with the parental behaviors in untreated CD38 knockout sires [22]. These results suggest that the central cholinergic pathway and NAcc neurons mediate this paternal behavior. The other brain regions that are involved remain to be elucidated.

**Figure 1:**
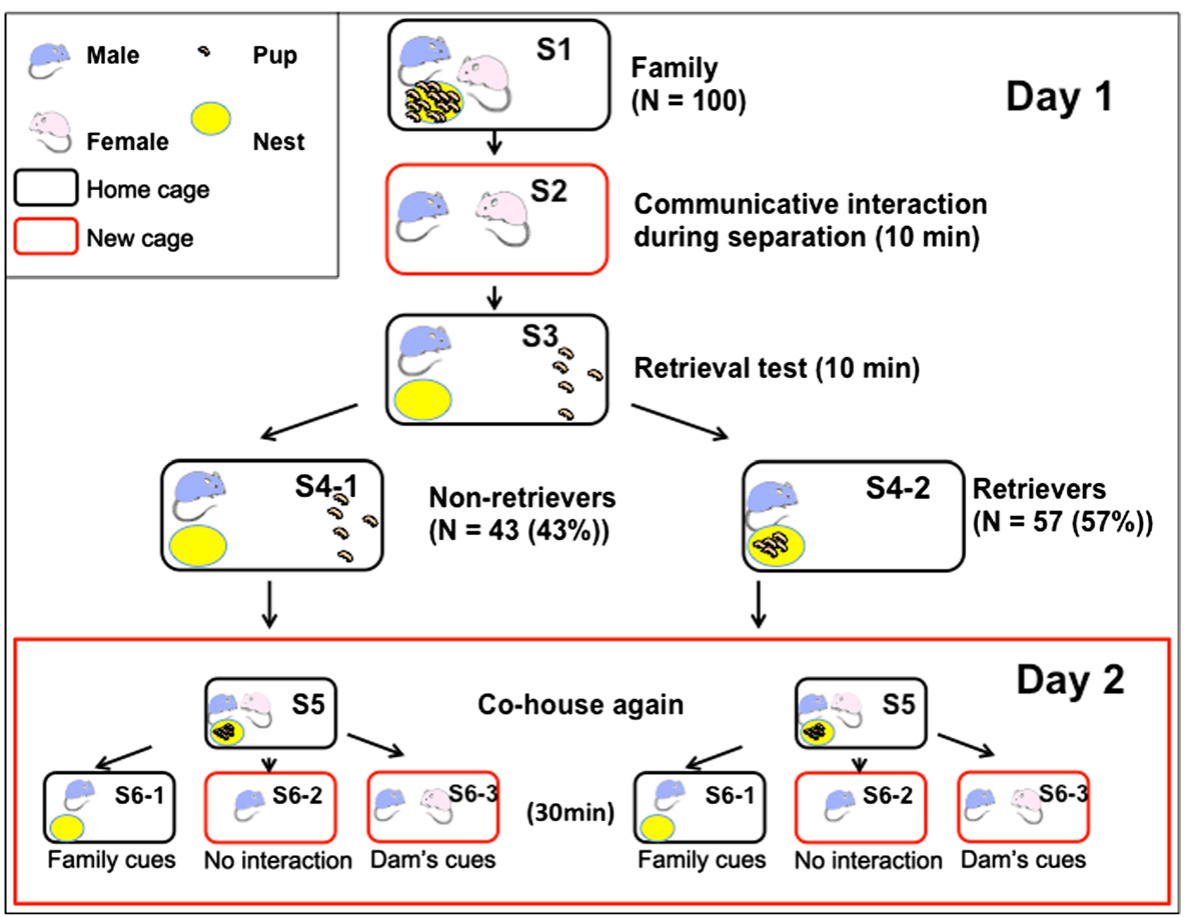
**Schematic drawings of the different housing conditions in the paternal behavior test.** On day 1, one male (blue) or female (pink) mouse and their biological pups were housed (S1 and S5) or isolated alone (S6-1 and S6-2) or together (S2, S6-3) in home (black) or new (red) cages. Then, retrieval was examined for five pups placed in a remote area from the nest (yellow) (S3). The pups in the nest represent a retrieval (S4-2), and those outside the nest represent no retrieval of pups to the nest (S4-1) by the sires, retrievers and non-retrievers, respectively. The number and percentage of sires represent the rate of retrieval or non-retrieval behaviors out of sires tested in S1. The retrieval-positive sires (retrievers) or retrieval-negative sires (non-retrievers) were then housed together with mates and pups as a family (S5). On the following day (Day 2), the two types of sires were separated in three different conditions (S6-1, −2 and −3). Each experiment had a 10-min duration in each step (S2, S3, S4). At the S6 step, the mice were maintained for 30 min before sacrifice.

**Figure 2:**
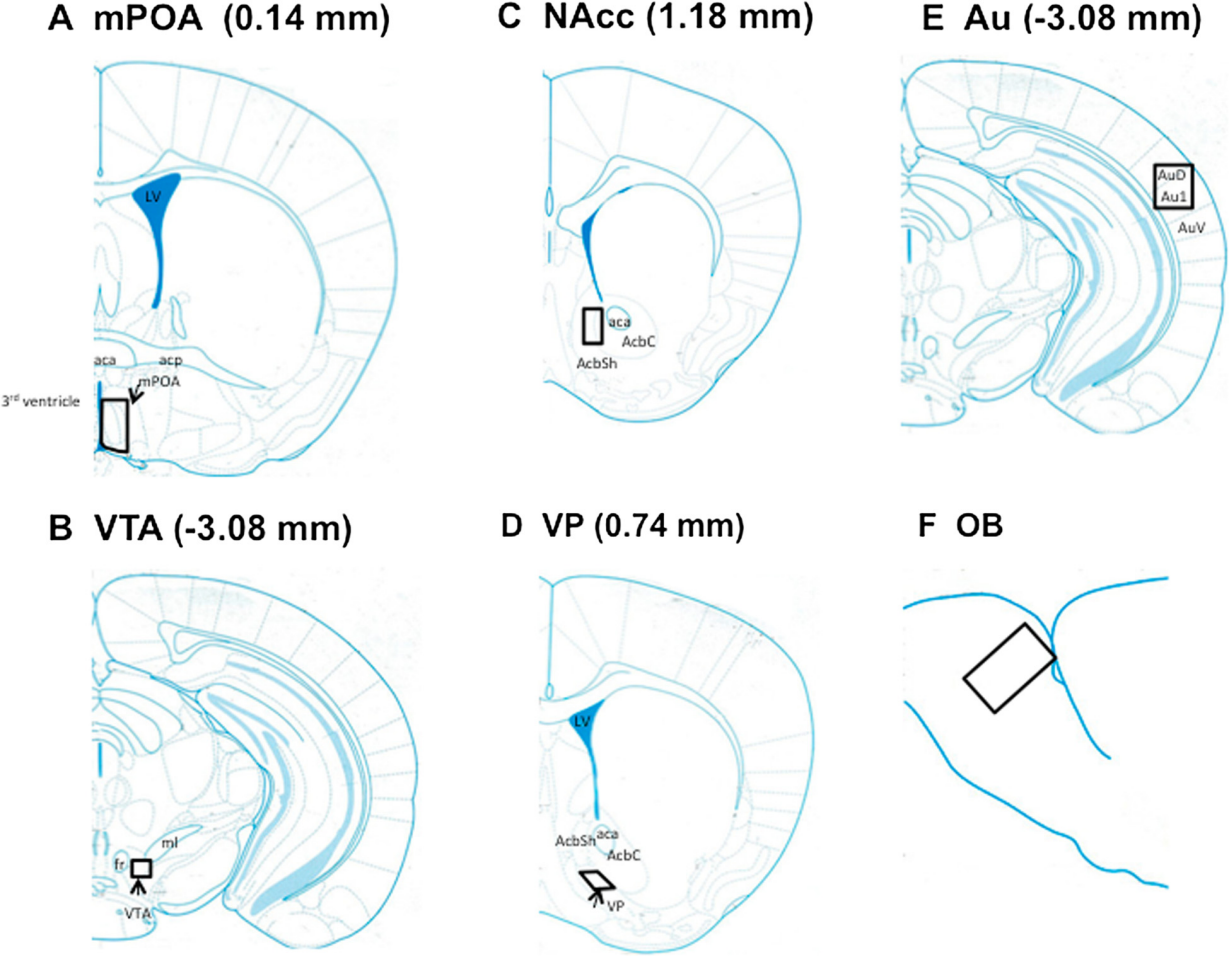
**Schematic representation of the brain regions analyzed for c-Fos immunoreactivity.** The distance from the bregma in the rostrocaudal plane is shown in parentheses in the medial preoptic area (mPOA, **A**), ventral tegmental area (VTA, **B**), nucleus accumbens (NAcc, **C**), ventral pallidum (VP, **D**), auditory cortex (Au, **E**) and olfactory bulb (OB, **F**) regions. Boxes represent the area histologically analyzed. The box in the mPOA covers subregions of the medial preoptic nuclei medial (100%) and lateral (50%), medial preoptic area (30%), medial preoptic area (30-40%) and anteroventral periventricular nucleus (100%). The drawings are coronal sections of the brain modified from Keith et al. [23]. LV, lateral ventricle; 3^rd^ ventricle, third ventricle; AsbSh, Shell of NAcc; AcbC, Core of NAcc.

**Figure 3:**
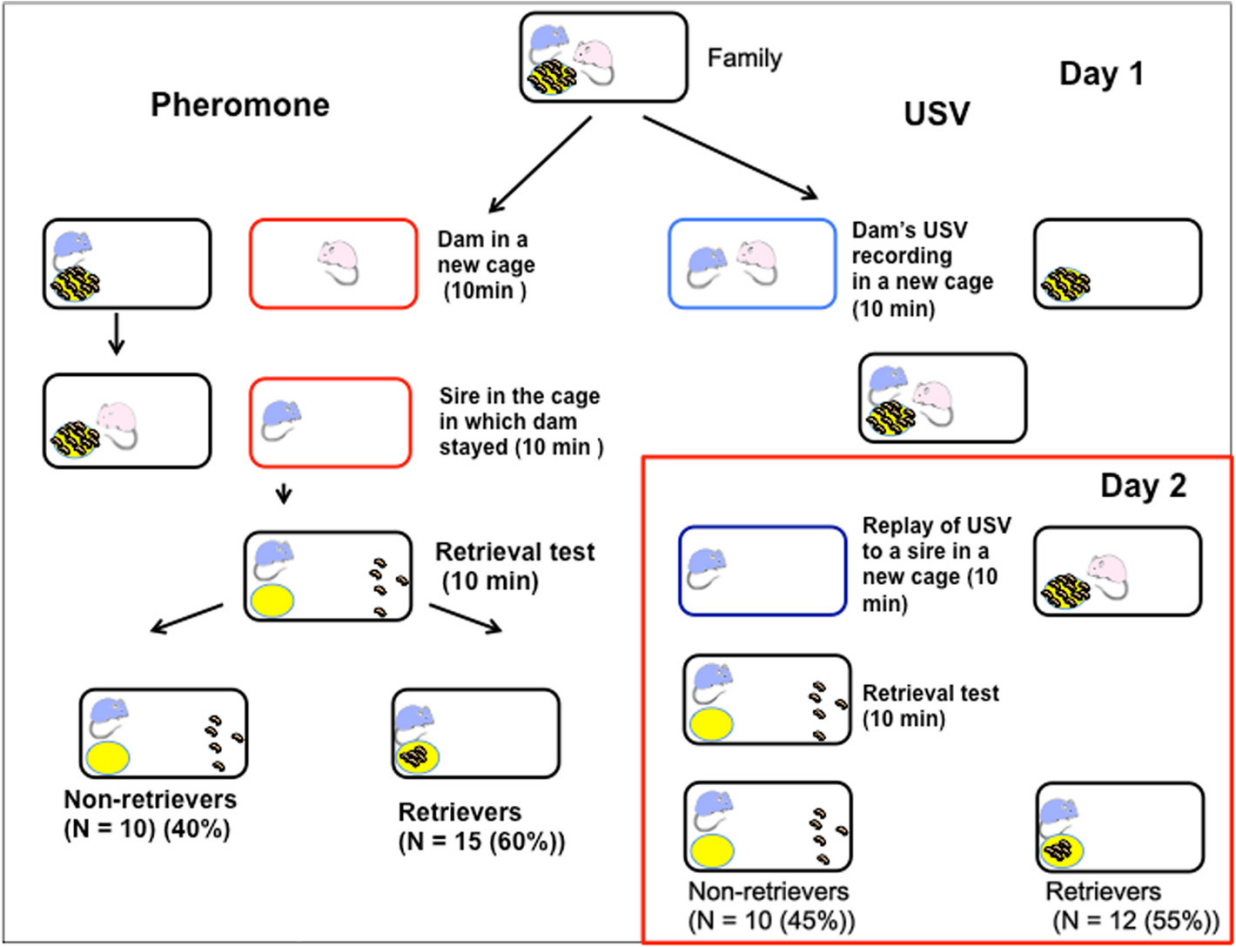
**Schematic drawings of the test to determine the effect of pheromones and ultrasound vocalization (USV) on the paternal behavior.** Left half: pheromones from a dam. A dam was placed in a new cage for 10 min. After removing the dam, the sire was immediately placed in the dam’s dirty cage for 10 min. After removal from the conditioned cage, the retrieval behavior of the sire was tested in the old home cage with 5 pups. The resulting number and percentage of retrievers and non-retrievers are indicated in parentheses. Right half: USVs were recorded in a new cage isolated together with the dam and sire for 10 min. On the following day, the USVs were replayed to the sire in another new cage for 10 min and retrieval behavior was tested in old cages. The resulting number of retrievers and non-retrievers are indicated in parentheses.

Behavioral responses toward pups by male mice appear to differ depending on the strain, the experimental conditions and the reproductive context [1]. Investigation of the paternal care neural circuit in each behavioral condition, i.e., in our mate-dependent care paradigm for ICR mice (Figure 1), is necessary [20]. Here, we quantified c-Fos protein, the product of proto-oncogene c-*fos* [3,4,10,11]. This method has been widely used as an indicator of trans-synaptic neuronal activation and has been adopted to investigate the participation of specific brain structures in the regulation of maternal behavior. Based on the hypothesis that the neural circuitry for maternal behavior proposed for females [18] is applicable to the neural circuitry of males, we examined the expression levels of c-Fos protein in the mPOA, VTA, NAcc and VP (at the indicated areas in Figure 2) of sires with or without the induction of paternal behavior by communicative interactions after co-housing between the mate pairs. We further studied the neural circuitry in the retrieval-positive or -negative sires by comparing two separation conditions (sire alone or co-housing), (Figure 1, Day 2) and the time course of c-Fos protein expression. Finally, the effects on c-Fos protein expression were examined by dissecting the signals of this communicative interaction, USV and pheromones (Figure 3).

## Results

### Experiments on day 1

Figure 1 illustrates the experimental paradigm of the different housing conditions of one family composed of a sire, dam and their biological pups and of the separation conditions for the induction of paternal behavior during a two-day experiment. In the first set of experiments, we performed pup-directed retrieval experiments (S1 – S4). We examined and compared the sires exhibiting retrieval (retrievers, S4-2) and those not exhibiting retrieval (S4-1, non-retrievers) during a 10-min observation period, after separation of the mated pair for 10 min (S3). The sires were sacrificed after an additional 10 min (a total duration of 30 min from exposure to the first social stimulation), allowing for c-Fos protein induction to a level that visible enough to for comparison, because a previous study [24] shows that c-Fos protein is expressed at 30 min but reaches peak at 90 min. We selected this time window to detect the effect due only to the co-housed condition, thus minimizing the influence of other factors, and a more preferred technique of an immunohistochemistry method to detect and quantify c-Fos protein.

**Figure 4:**
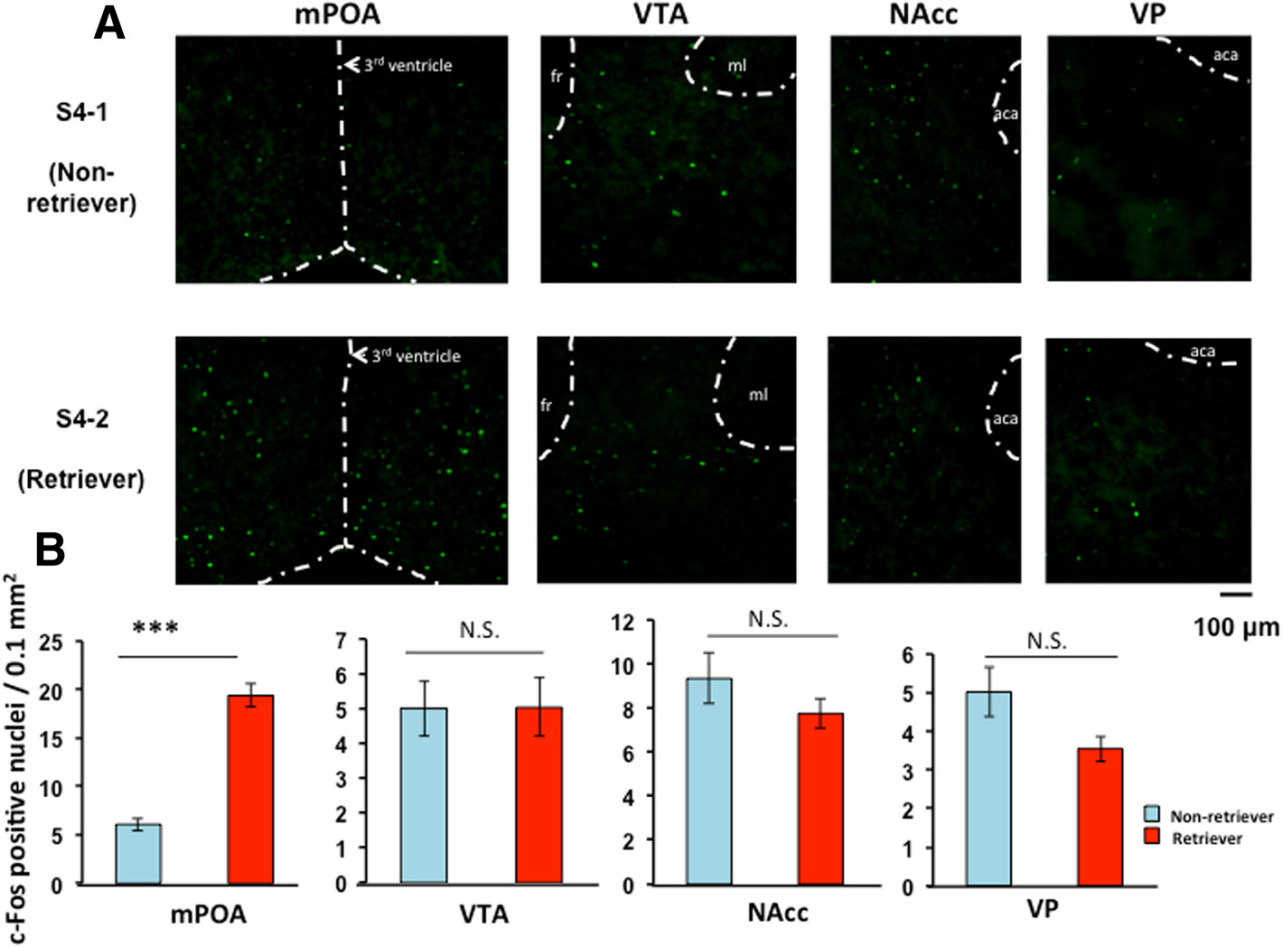
**Photomicrographs of coronal sections showing c-Fos-immunoreactivity. (A)** Representative images of c-Fos in the mPOA, VTA, NAcc, and VP regions of non-retrievers (S4-1) and retrievers (S4-2). The brains were fixed approximately 30 min from the start of the separation experiments (stimulation, S2). **(B)**. The data of the number of c-Fos-positive cells are expressed as the mean ± s.e.m. (n = 12 in each area); ***P < 0.0001, Two-tailed, Student’s *t*-test. N.S., not significant.

The number of mPOA c-Fos-immunoreactive cells (Figure 4A) in the retrievers was significantly higher than that of the non-retrievers. The mean percentage increase of c-Fos positive cells in the 0retrievers was calculated as: (the cell number in each retriever)/(average cell number in the non-retrievers) × 100 and was 320 ± 19.4% for mPOA (n = 12, *P* = 0.0000, two-tailed Student’s *t*-test; Figure 4B). Expression of c-Fos in the VTA, NAcc and VP in retrievers was neither higher nor lower when compared with non-retrievers (Figure 4). These results indicate that c-Fos expression is significantly higher only in the mPOA of retrievers and suggest that the mPOA is a responsive region for paternal retrieval behavior, which is consistent with the findings for maternal behavior [12,18].

### Experiments on day 2

**Figure 5:**
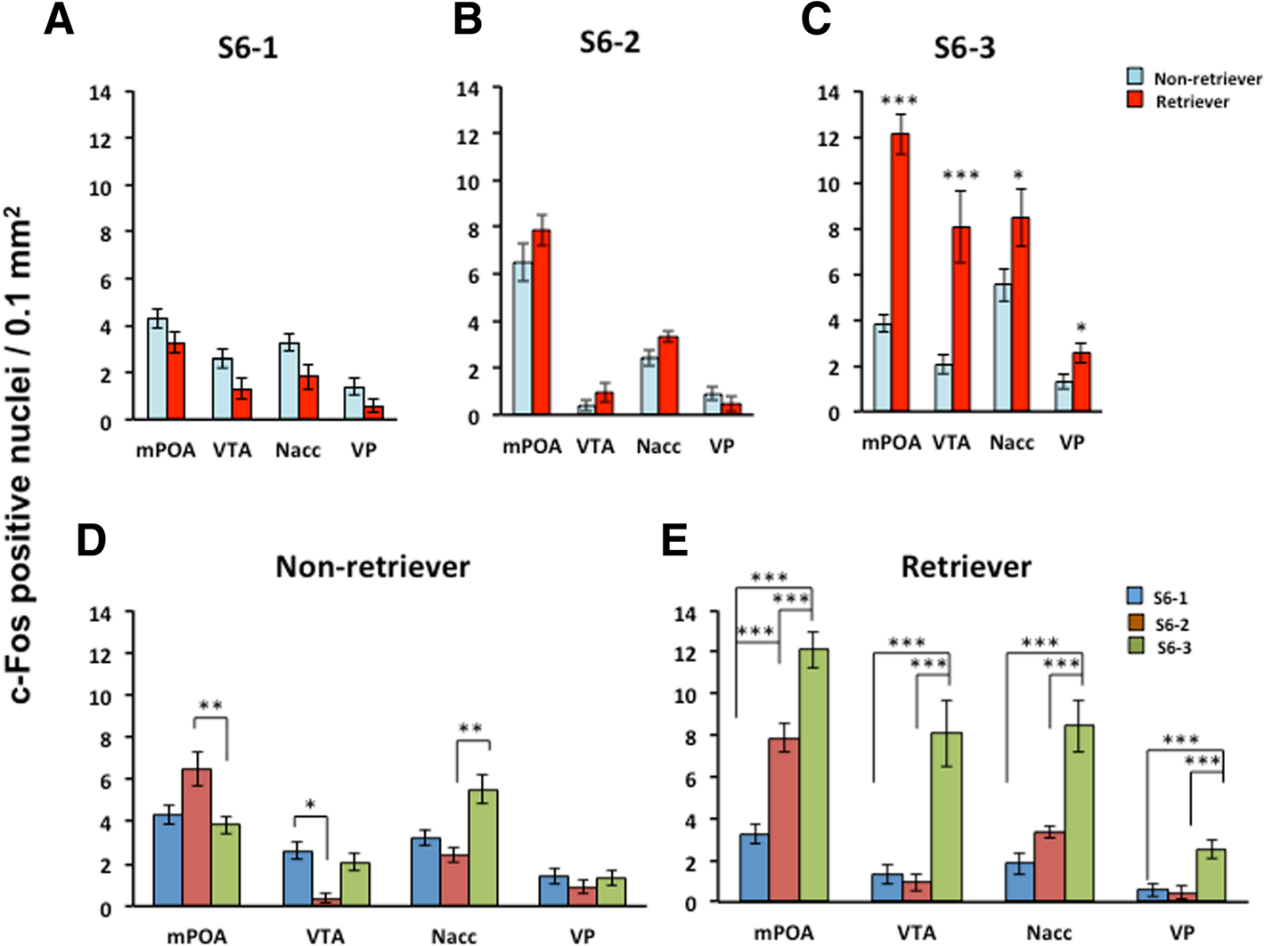
**c-Fos expression in sires on day 2.** The number of c-Fos-positive cells in the four brain regions of the sires from photomicrographs of coronal sections of c-Fos immunoreactivity at 30 min from the various separation conditions. The sires tested were in the retrieval-negative (derived from S4-1) and -positive families (derived from S4-2). The sires of both retriever and non-retriever types were isolated in old cages alone (S6-1, **A**), in new cages alone (S6-2, **B**) or together with his mate in new cages (S6-3, **C**). Statistical analysis of two-way ANOVA is listed in Table 1; Significant differences were found between non-retrievers and retrievers at *P < 0.05 and ***P < 0.001, respectively, two-way Student *t*-test, n = 4. The above data **(A-C)** of the quantity of c-Fos positive cells in three conditions (S6-1 to S6-3) at different brain regions are replotted according to two groups of non-retrievers **(D)** or retrievers **(E)**, One-way ANOVA analysis followed by Bonferroni’s *post-hoc* test for retrievers **(E)** reveals the following: mPOA, *F*_2,21_ = 42.11, *P* = 0.0000; VTA, *F*_2,21_ = 17.00, *P* = 0.0000; NAcc, *F*_2,20_ = 17.33, *P* = 0.0000; VP, *F*_2,21_ = 10.80, *P* = 0.0006; n = 4 for each case. Significant differences at **P < 0.01 and ***P < 0.001, respectively.

In the second set of experiments, we compared the efficacy of social situations (isolation alone vs. co-housed separation) on c-Fos activation in identical groups, composed of sires with either retrieval or non-retrieval tendencies. After the retrieval experiments (S1 – S4 in Figure 1), on experimental day 1, we selected the families with sires that exhibited retrieval behavior (S4-2, 56% out of 100 sires tested) and those that did not (S4-1, 44%). Then, the families were allowed to live as an intact family for 24 h (S5). On the following day, the sires were isolated alone in the old cage with family cues (S6-1) or in a new cage with no cues (S6-2) with the parents together (communicative interaction between the couples, S6-3) after being isolated from the pups and placed in each cage for 10 min. The quantity of c-Fos-positive cells in the four tested brain regions was compared between non-retriever (S4-1-families) and retriever (S4-2-families) sires among S6-1, S6-2 and S6-3 conditions (Figure 5A-C). The number of cells that were positive for c-Fos protein was significantly higher in the retrievers than in the non-retrievers in all of the brain regions tested under the co-housed condition (S6-3, Figure 5C; two-way ANOVA for retrieval behaviors and conditions is shown in Table 1; Significant differences were found between non-retrievers and retrievers at *P < 0.05 and ***P < 0.001, respectively, two-way Student *t*-test). Such increases in the retrievers were not observed in the other two conditions (S6-1 and S6-2, Figure 5A and B).

**Table 1:** **c-Fos expression in sires on day 2**

mPOA	Behavior	F_1,68_ = 25.83	P = 0.0000
	Condition	F_2,68_ = 19.42	P = 0.0000
	Behavior and condition	F_2,68_ = 25.25	P = 0.0000
VTA	Behavior	F_1,65_ = 10.27	P = 0.0021
	Condition	F_2,65_ = 23.70	P = 0.0000
	Behavior and condition	F_2,65_ = 18.02	P = 0.0000
NAcc	Behavior	F_1,59_ = 1.38	P = 0.2455
	Condition	F_2,59_ = 19.47	P = 0.0000
	Behavior and condition	F_2,59_ = 3.49	P = 0.0368
VP	Behavior	F_1,69_ = 0.00	P = 0.9544
	Condition	F_2,69_ = 5.99	P = 0.0040
	Behavior and condition	F_2,69_ = 4.10	P = 0.0208

Data are the statistical analysis results obtained with two-way ANOVA (corresponding to Figure 5).

Behavior means no-retrieval and retrieval.

Condition means S6-1, S6-2 and S6-3.

Alternative comparisons are shown in Figure 5D and E. The number of c-Fos positive cells was the highest in the S6-3 conditions among three isolation conditions in retrievers (Figure 5E), compared to non-retrievers (Figure 5D). Significant differences were found between S6-1 *vs* S6-2, S6-1 *vs* S6-3, or S6-2 *vs* S6-3 at *P < 0.05 and ***P < 0.001, respectively, two-way Student *t*-test. These data show that the mPOA-VTA-NAcc-VP circuit in retriever sires is likely to be sensitized by the dams’ communicative interaction.

**Figure 6:**
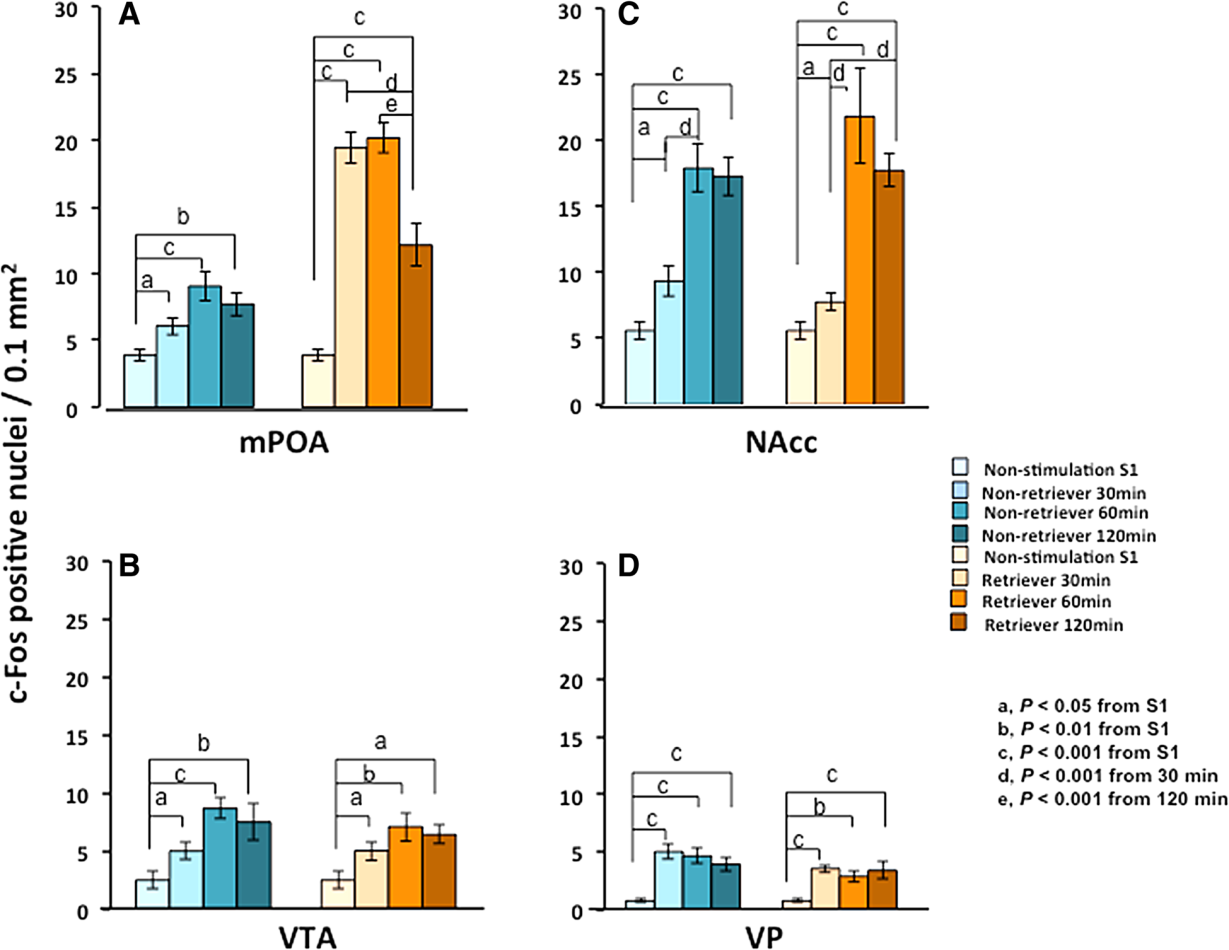
**Time course of c-Fos expression.** The numbers of c-Fos-positive cells in the four brain regions (mPOA **(A)**, VTA **(B)**, NAcc **(C)** and VT **(D)**, respectively) of the sires were counted from photomicrographs of coronal sections of c-Fos immunoreactivity with a time window of 30, 60 and 120 min from the start of separation in new cages together with the mate dam. The sire just before isolation (the S1 condition) was used for time zero. Sires in the nonretriever (blue bars, S4-1) and retriever (brown bars, S4-2) families. Statistical analysis of two-way ANOVA is listed in Table 2. One-way ANOVA followed by Bonferroni’s *post-hoc* test revealed significant differences between non-stimulation and each time point at ^a^ *P* < 0.05, ^b^ *P* < 0.01, ^c^ *P* < 0.001 from S1; ^d^ *P* < 0.001 from 30 min; ^e^ *P* < 0.001 from 120 min, respectively.

Finally, we examined the time dependency of c-Fos expression. At 30, 60 and 120 min from the start of stimulation (male sires placed in a new cage with mate dams) or before the cohousing condition (S-1), the number of c-Fos-positive cells increased with time in both the non-retrieval and retrieval groups (Figure 6A-D). Two-way ANOVA also revealed that there is a significant enhancement in retrievers than non-retrievers only in the mPOA region (Table 2). Interestingly, the maximal level of the increased c-Fos positive cell number was already obtained at 30 min in the mPOA in retrievers. (One-way ANOVA followed by Bonferroni’s *post-hoc* test revealed significant differences between non-stimulation and each time point at ^a^*P* < 0.05, ^b^*P* < 0.01, ^c^*P* < 0.001 from S1; ^d^*P* < 0.001 from 30 min; ^e^*P* < 0.001 from 120 min, respectively).

**Table 2:** **The statistical analysis results on time course of c-Fos expression obtained by two-way ANOVA (corresponding to Figure** 6 **)**

mPOA	Behavior	F_1,139_ = 111.70	P = 0.0000
	Time	F_3,139_ = 56.02	P = 0.0000
	Behavior and time	F_3,139_ = 22.57	P = 0.0000
VTA	Behavior	F_1,113_ = 0.85	P = 0.3598
	Time	F_3,113_ = 14.21	P = 0.0000
	Behavior and time	F_3,113_ = 0.37	P = 0.7768
NAcc	Behavior	F_1,104_ = 0.30	P = 0.5865
	Time	F_3,104_ = 32.07	P = 0.0000
	Behavior and time	F_3,104_ = 0.87	P = 0.4598
VP	Behavior	F_1,130_ = 6.40	P = 0.0126
	Time	F_3,130_ = 22.67	P = 0.0000
	Behavior and time	F_3,130_ = 1.44	P = 0.2345

Behavior means no-retrieval and retrieval.

Time means the sire was sacrificed at zero, 30, 60 and 120 min from the start of separation in new cage together with the mate dam (from S2 to S4). The sire just before isolation (the S1 condition) was used for time zero.

### c-Fos expression after retrieving tests by USVs and pheromones in the mPOA

**Figure 7:**
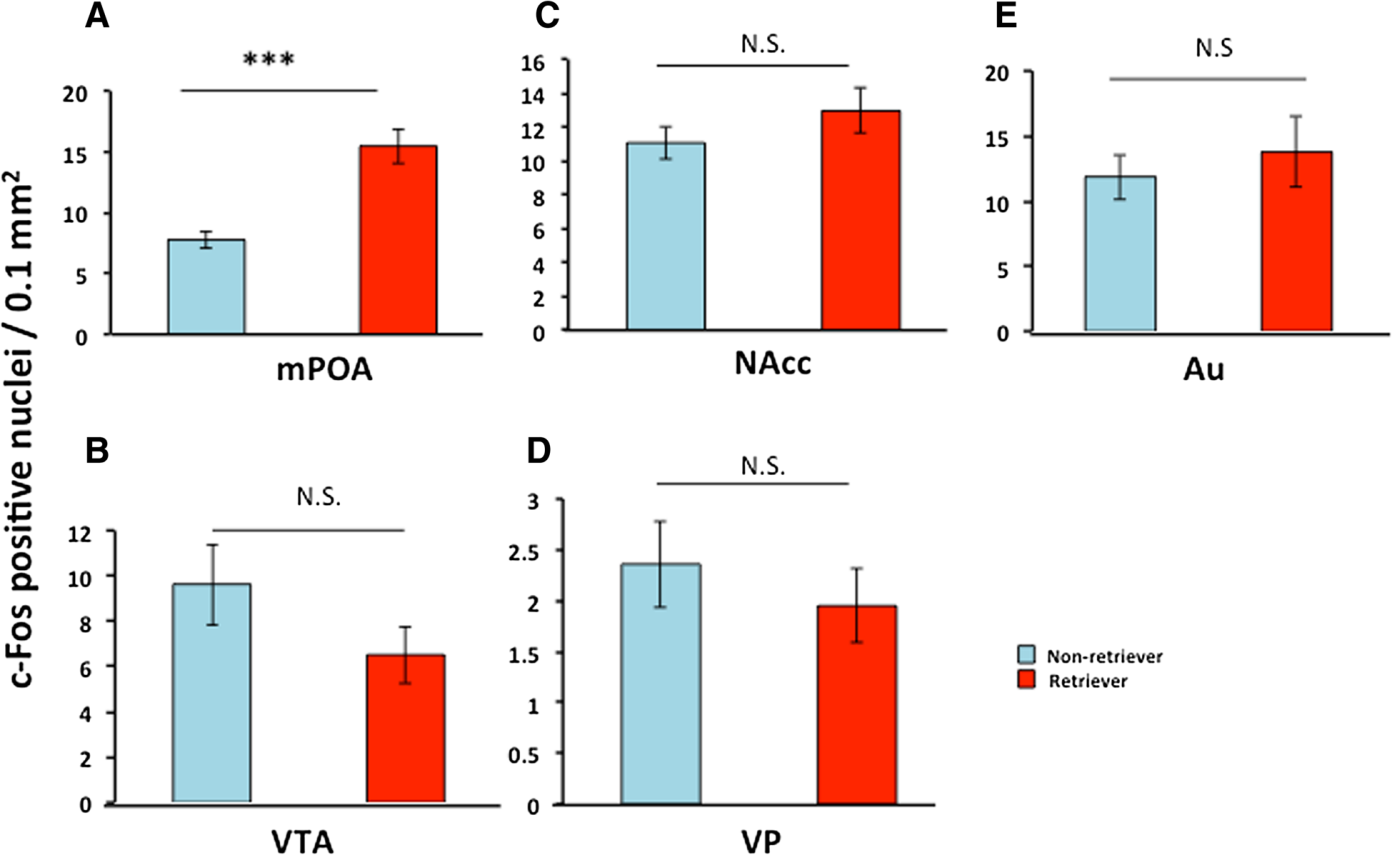
**c-Fos expression in retrieval-positive and -negative sires after stimulation with dam’s USV.** Representative images of c-Fos-positive cells are shown in the mPOA **(A)**, VTA **(B)**, NAcc **(C)**, and VP **(D)** regions and the auditory cortex **(E)**. The brains were fixed for approximately 30 min from the start of the separation experiments stimulated with replaying of dam’s USVs and the retrieval test. The data are expressed as the mean ± s.e.m. (n = 6 each); ***P < 0.0001, Two-tailed, Student’s *t*-test. N.S., not significant.

We examined the effects of two known factors of communicative interactions, USV and pheromones. As shown in Figure 3, we stimulated a sire by isolation in a new cage placed in a soundproof box. The USVs recordings of his mate that were made one day earlier were played from a loud speaker for 10 min. The sire was examined for his retrieval behavior at day 2. Thirty min after the start of USV stimulation and the retrieval test, we scarified and harvested the brain and assessed the c-Fos protein expression. The average number of c-Fos positive cells in the mPOA was significantly higher in the retrievers than in the non-retrievers (Figure 7A): 199.5 ± 18.3% of the non-retriever’s value, n = 6, *P* = 0.0000, two-way Student's *t*-test. No significant difference was observed in the VTA, NAcc and VP regions (Figure 7B-D), including the auditory cortex (Figure 7E).

**Figure 8:**
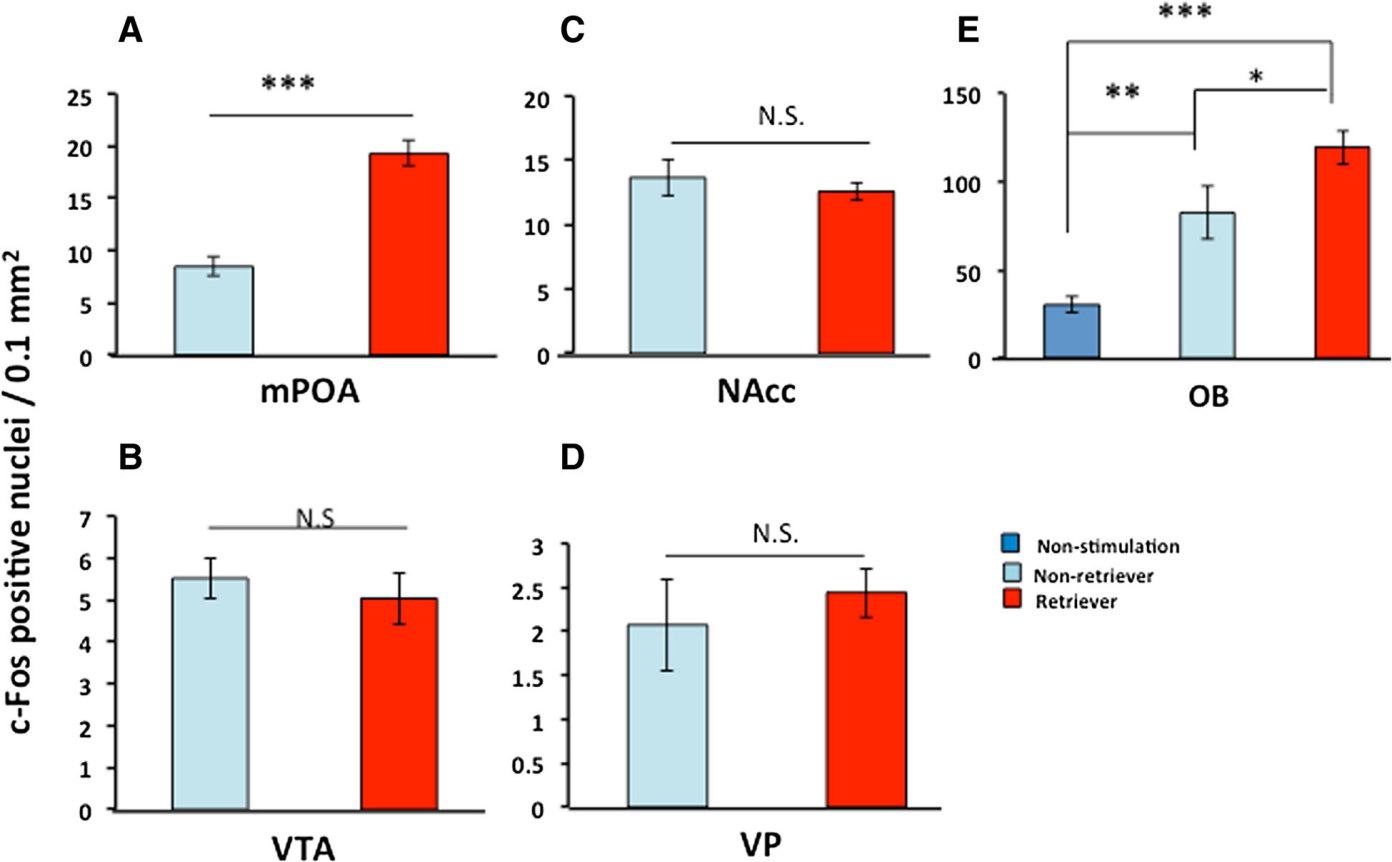
**c-Fos expression in retrieval-positive and -negative sires after stimulation with dam’s pheromones.** Representative images of c-Fos-positive cells are shown in the mPOA **(A)**, VTA **(B)**, NAcc **(C)**, VP **(D)**, and olfactory bulb (OB, **E**) regions. The brains were fixed for approximately 30 min from the start of the separation experiments in the dam’s conditioned cages and the retrieval test. The data are expressed as the mean ± s.e.m. (n = 6 each); ***P = < 0.0001, Two-tailed, Student’s *t*-test.

The effect of dam pheromones was examined by placing a sire in a cage used by his mate for 10 min immediately preceding the experiment; the dam was removed from the cage, and the sire was transferred to this cage for 10 min, during which the conditioned cage exhibited the pheromones of the dame (Figure 3). After identification of retrievers and non-retrievers at 30 min from the start of pheromonal experiments, the mouse brain was harvested to assess the c-Fos protein expression. The average number of c-Fos positive cells in the mPOA were higher in the retrievers than in the non-retrievers (Figure 6A): 227 ± 15.0% of the non-retriever’s value, n = 6, *P* = 0.003, two-way Student’s *t*-test. No difference was observed in the VTA, NAcc and VP (Figure 8B-D). In the olfactory bulb, the number of c-Fos positive cells was higher in retrievers than non-retrievers (Figure 8E), while expression levels were significantly higher than in unstimulated sires (such as in the S1 condition): one-way ANOVA followed by Bonferroni’s post-hoc test: *F*_2,34_ = 19.77, *P* = 0.0000, *P* = 0.002, *P* = 0.000, *P* = 0.051, n = 6, respectively, from unstimulated mice. These results indicate that USV and pheromones, two independent components of communicative interaction between sires and dams, are effective factors for mPOA activation. Olfactory stimulation activated olfactory neurons, while auditory stimulation generated no difference between the two types of sires, retrievers and non-retrievers.

## Discussion

These results demonstrate that contrasting the retriever and non-retriever sires allowed us to determine the effect of a single retrieval session on mPOA activity, a region known to regulate pup-induced parental behavior [2,5,11]. Our result is consistent with data showing the critical role of the mPOA in pup-induced maternal and paternal behavior in mice and other mammals [2–17].

The second result in this study is that c-Fos immunoreactivity was increased in the mPOA region more in retriever sires than non-retrievers that received 38-kHz USV or unidentified pheromones from their mate dams. Because both stimuli directly increased c-Fos protein under no interaction with pups (Table 3), to our knowledge, this study is the first to report that c-Fos activation in the neurons of sires is potentially triggered by interactive communicative factors with dams instead of with pups. There is no difference between retrievers and non-retrievers in c-Fos expression in the auditory cortex following USV stimulation (Figure 5E), but a significant difference in the olfactory bulb by pheromones (Figure 8E). Therefore, it is necessary to investigate how the primary regions of the signal inputs are differentially activated by these stimuli.

**Table 3:** **c-Fos-positive cells in the mPOA in sires with or without stimulation of USV or pheromones under no influence of pups**

**Condition**	**c-Fos positive nuclei/0.1 mm** ^**2**^	**(n)**
No stimulation	7.0 ± 0.32	(4)
USVs	17.3 ± 1.95***	(4)
No stimulation	6.2 ± 0.7	(5)
Pheromones	13.9 ± 1.22**	(5)

The brains of experimentally naïve sires were fixed approximately 30 min from the start of exposing to USVs or no sound for 10 min in new cages and of placing in dam’s dirty cages or new empty cages for 10 min. The sires’ retrieval ability was not examined before a sacrifice.

The data of the number of c-Fos-positive cells are expressed as the mean ± s.e.m. in n pairs. **P < 0.01, ***P < 0.001, Two-tailed, Student’s *t*-test.

Expression of c-Fos induced by exposure to pups has been reported in other brain regions of interest that are associated with vole paternal behavior [11], including the medial nucleus of the amygdala, the lateral septum, the medial bed nucleus of the hypothalamus and the accessory olfactory bulb. In rats, experimental evidence has revealed that some of these brain regions are involved in promoting the expression of maternal behavior [2,11,19]. Whether c-Fos expression is induced by communicative interaction in the above mentioned brain regions should be determined.

Our third conclusion from the second day of experiments is that the number of cells expressing c-Fos in the S6-3 condition is higher than those in the S6-2 condition, with average increases by communicative interaction over no interaction ((cell density in S6-3)/(cell density in S6-2) × 100) of: 154 ± 45% (*P* = 0.000), 860 ± 23% (*P* = 0.000), 253 ± 41% (*P* = 0.000) and 555 ± 13% (*P* = 0.000) in the mPOA, VTA, NAcc and VP regions, respectively (Figure 5B and C). This increase appears to be a net difference caused by the presence of the mate dam in the cage together with the sire, although transferring to new cages condition (environment change) was equal. However, our previous report showed that retrieval behavior is usually observed in the S6-1 condition [20]. If we assume that the induction of c-Fos in the mPOA is responsible for paternal behavior, c-Fos expression should be higher in the mPOA. The reason for this discrepancy is unclear at this moment. This point should be examined in the near future.

Approximately 30 to 60 min is required to achieve peak mRNA expression of *c-fos* and 90–120 min is required to achieve peak c-Fos protein expression after exposure to a stimulus [24–28]. We examined c-Fos protein expression approximately 30 min after the start of stimulation in most of the current experiments, because quantification was applicable with relatively high accuracy by the current (Metamorph) software. We selected this short duration to reflect the direct interaction between pairmates by controlling environment-related effects on brain neuronal activity. To support using the 30 min duration, we examined c-Fos protein induction after 30, 60, and 120 min by leaving the sires in the retrieval test condition (S4-1 and S4-2). Although the number of c-Fos positive cells was high at 60 min, a duration of 30 min was sufficient to induce expression in two regions, and a duration of 120 min resulted in a slight decline (Figure 6, brownish color bars). Furthermore, the mean percentage increase of the c-Fos positive cell number in the retrievers over that in the non-retrievers was relatively unchanged at each time point: Two-way ANOVA for retrieval behaviors and time course (Table 2). Thus, these results may allow for a comparison of c-Fos levels at the 30-min time duration.

To compare c-Fos protein levels before and after exposure to stimuli using the protocol by Numan and Numan [28], where the subject mother or father rats were separated from pups for two days to achieve complete degradation of c-Fos protein, requires >6 hours. In our experiments, we were unable to use this protocol to reduce c-Fos because any changes in the family situation altered the behavior of the sires. Although we were unable to determine the basal c-Fos level of the sires from which induction of c-Fos may occur, our method was able to measure c-Fos levels at the S1 condition before the start of the S2 condition. The level at S1 was significantly lower than S4 condition.

## Conclusions

It is likely that the mPOA-VTA-NAcc-VP neural circuit in sires induces paternal behavior, although we could not determine the circuit sequence. Further studies are required to detail the role of this neural circuit in the regulation of paternal behavior in mice under this paradigm.

## Methods

### Animals

Male and female Slc:ICR mice were obtained from Japan SLC, Inc. (Hamamatsu, Japan) via a local distributor (Sankyo Laboratory Service Corporation, Toyama, Japan). The offspring of these mice were born in our laboratory colony and housed until pairing. The animals were paired and kept in our laboratory under standard conditions (22°C; 12-h light/dark cycle, lights on at 8:45 a.m.) in standard mouse cages (300 mm × 160 mm × 110 mm), with sawdust bedding, with as food and water provided *ad libitum*, as previously described [20,29]. All of the animal experiments were approved and performed in accordance with the Fundamental Guidelines of the Committee on Animal Experimentation of Kanazawa University.

Virgin males and females were paired at 45–55 d and continuously housed together in a standard mouse maternity cage until after the delivery of pups on postnatal day 3–5. The average litter size of the dams was 13.5 ± 0.67 (n = 30) [22]. Each of the family units, composed of the new sire and dam and their first litter, were experimentally naïve and used once (Figure 1, S1).

The parents were isolated from the pups to treat them in new cages (environments) for 10 min [20] (S2), in which we detected communicative interaction from the dam to the sire via 38-kHz ultrasonic vocalization and unidentified pheromones [20,21]. Five pups were randomly selected from the litter and placed individually at a site remote from the nest in the original family cage. After 10 min in the new cage, each sire was returned to its home cage that housed his five biological pups to assess parental retrieval behavior (S3). The percentage of sires exhibiting retrieval behavior was quantified during the 10 min following reunion with the pups (S4-1 or S4-2). The sires that carried all five of their pups to the original nesting place or within two thirds of the distance between the nest and the new location were defined as retrieval-positive or retrievers (S4-2) [20,30]. The sires that did not fulfill the above behavioral definition were designated as retrieval-negative or non-retrievers (S4-1). The behavioral tests were conducted at 10:00–15:00 h in a randomly mixed sequence of experimental groups.

In the second set of experiments, we performed retrieval experiments, as above (S1 – S4), and selected the families with the sires exhibiting retrieval (S4-2). The following day, the parents (S5) were isolated from the pups under one of the following conditions: 1) the sire was left alone in the family cage, in which family cues were present, without direct interaction with his mate (S6-1); 2) the sire was placed alone without communicative interaction in a new environment (S6-2); or 3) the sire and dam were co-housed under communicative interaction in a new environment (S6-3).

### Immunohistochemistry

After each experiment, the sires were anesthetized, sacrificed and prepared for c-Fos immunohistochemistry. The mice were intracardially perfused with cold PBS followed by a cold 4% paraformaldehyde (PFA) in phosphate-buffered saline (PBS) solution. The brains were removed and post-fixed in a 4% PFA solution overnight at 4°C. Brain regions were cut into two to four larger blocks. The blocks were sliced on a microtome into 20-μm−thick sections. The sections were pre-incubated in blocking solution (3% bovine serum albumin and 0.3% Triton X-100 in PBS) for 1 h, then incubated with an anti-c-Fos antibody (sc-52, 1:200; Santa Cruz Biotechnology, Santa Cruz, CA, USA) in blocking solution for 12 h at 4°C. After three washes with washing buffer, the sections were incubated with goat anti-rabbit IgG antibody coupled with Alexa Fluor 488 (Invitrogen, Carlsbad, CA, USA) in blocking solution for 1 h at room temperature. The images were obtained using an Olympus IX71 inverted microscope equipped with a cooled CCD camera (Cool SNAP HQ2; Roper Scientific, Tucson, AZ, USA). The number of c-Fos immuno-positive nuclei in each brain section was recorded and analyzed using Metamorph software (Molecular Devices, Downingtown, PA, USA), as described previously [31,32]. Only the c-Fos-positive nuclei within a specific size range were counted and a constant threshold level of staining was used to distinguish c-Fos-positive cells as follows: a fluorescence diameter < 13.5 μm and intensity above 445.6 (arbitrary units) were counted. The average fluorescence intensity was within the 5-fold range (Figures 2, 3 and 4).

The distance from the bregma in the rostrocaudal plane is 0.14 mm for the mPOA, −3.08 mm for the VTA, 1.18 mm for the NAcc and 0.74 mm for the VP, according to Keith et al. [23]. The locations of the four brain regions and two primary sensory brain areas [33] are specified in the lower magnification diagram in Figure 2.

### Statistical analysis

Two-tailed Student’s *t*-test was used for the single comparisons between the two groups. The remaining data were analyzed using one-way or two-way analyses of variance (ANOVA). *Post-hoc* comparisons were performed only when the main effect was statistically significant. The *P*-values for multiple comparisons were adjusted using the Bonferroni correction.
